# Real-Time Perfusion and Leak Assessment in Bariatric Surgery: Bridging Traditional and Advanced Techniques

**DOI:** 10.7759/cureus.71919

**Published:** 2024-10-20

**Authors:** Amir Farah, Anna Tatakis, Kamil Malshy, Ahmad Mahajna, Sa'd Sayida

**Affiliations:** 1 Surgery, Medical College of Wisconsin, Milwaukee, USA; 2 General Surgery, Medical College of Wisconsin, Milwaukee, USA; 3 Urology, University of Rochester Medical Center, Rochester, USA; 4 General Surgery, Division of Advanced Laparoscopic and Bariatric Surgery, Rambam Medical Center, Haifa, ISR; 5 General Surgery, Edinburgh Medical Missionary Society (EMMS) Nazareth Hospital, Nazareth, ISR

**Keywords:** anastomosis, bariatric surgery, hsi, icg, leak, leak test, lsci, obesity, roux en y gastric bypass, sleeve gastrectomy

## Abstract

This comprehensive literature review explores the efficacy of real-time perfusion and leak assessment methods in bariatric surgery, comparing traditional techniques with advanced imaging modalities. As the global incidence of obesity and related comorbidities rises, the demand for bariatric surgeries such as Roux-en-Y gastric bypass and sleeve gastrectomy has increased, along with the risk of serious complications like anastomotic and staple line leaks. Traditional intraoperative leak testing methods, including the air leak and methylene blue dye tests, are commonly employed but exhibit inconsistent sensitivity in leak detection. Intraoperative endoscopy, although underutilized, offers enhanced visualization and has been associated with reduced leak and complication rates in certain cases. Emerging technologies such as indocyanine green (ICG) fluorescence, laser speckle contrast imaging (LSCI), and hyperspectral imaging (HSI) provide real-time assessment of tissue perfusion, potentially improving surgical outcomes. ICG fluorescence enables visualization of blood flow to detect ischemia, while LSCI offers immediate, dye-free perfusion mapping, and HSI assesses tissue oxygenation without the need for contrast agents. Despite their promise, these technologies are limited by high costs, technical complexity, and varying accessibility, with current evidence insufficient to confirm their superiority over traditional methods. Future research should focus on large-scale, multicenter trials to validate these advanced techniques and refine their application in clinical practice. Integrating traditional and emerging methods may optimize intraoperative decision-making, reduce complications rates, and enhance patient outcomes in bariatric surgery.

## Introduction and background

As obesity rates and obesity-related comorbidities (cardiovascular disease, type 2 diabetes mellitus, and cancer) continue to rise, so does the number of bariatric procedures worldwide [1]. Metabolic bariatric surgery remains the most effective treatment for morbid obesity and has been shown to decrease cardiovascular disease events and deaths due to certain cancers and type 2 diabetes mellitus, in addition to decreased all-cause mortality [2,3]. The increasing number of operations inevitably increases the number of overall complications, the most feared of which are anastomotic and staple line leaks [4].

The most commonly performed bariatric surgeries in the United States are sleeve gastrectomy (SG) and Roux-en-Y gastric bypass (RYGB) [5]. Published leak rates for these surgeries range from 0% to 8% for SG and 0.1-8.3% for RYGB [6-11]. Several factors, such as ischemia, mechanical failure, anatomic considerations, inflammation, and patient-related factors, have been implicated in the pathogenesis of a leak, although some also suggest a multifactorial cascade [12]. A proposed classification system for leaks was described by Csendes et al. in 2010, which distinguishes between three categories of leaks based on timing [13]. Early leaks occur within one to four days postoperatively; intermediate leaks occur between five and nine days; and late leaks present 10 or more days after surgery [13].

Intraoperative leak testing (IOLT) has been proposed as a strategy to reduce undetected leaks during bariatric surgery, although its effectiveness remains controversial and its routine use in RYGB and SG has yielded mixed results in the literature [14-16].

This review aims to explore the current literature on IOLT in RYGB and SG, focusing on its methods, effectiveness, and potential advantages and drawbacks.

## Review

Traditional methods of IOLT

Several techniques are employed for IOLT, with the air leak test and methylene blue dye test being the most commonly used [17]. Air leak testing is performed by submerging the gastric pouch or the anastomosis in saline while insufflating air through an orogastric tube or an endoscope and observing for air bubbles [18]. Alternatively, the methylene blue dye test involves flushing methylene blue through an orogastric tube, and any dye extravasation along the staple line or anastomosis indicates a leak, enabling immediate repair [19].

Both the air leak test and the methylene blue dye test provide relatively straightforward methods for detecting leaks intraoperatively. Their clinical value seems to be their ease of use. However, their reliability in identifying ischemic leaks may be limited, highlighting the need for advancements in IOLT technology.

Intraoperative endoscopy (IOE) is another technique utilized during RYGB and SG to assess the integrity of the staple line or anastomosis [20]. Some bariatric surgeons advocate for its routine use, as it has been linked to a reduced incidence of leaks, bleeding, and stenosis [21]. IOE is particularly valuable in SG, where endoscopy can be employed to detect a predisposition to stenosis due to twisting or narrowing of the remaining stomach, which would also increase the risk of a leak [20]. This proactive approach allows surgeons to identify and correct anatomical issues intraoperatively, aiming to improve overall outcomes.

Despite its growing adoption, IOE employment varies widely among surgical centers, possibly due to concerns over increased procedural time, lack of training among surgeons, and resource costs, although more studies are needed to validate these points.

Benefits of IOLT

One of the key advantages of IOLT is the early detection of leaks when the IOLT is positive, allowing surgeons to address the leak while still in the operating room [22]. The intraoperative repair of these intraoperatively diagnosed leaks led to a 60% reduction in clinically significant leaks in some series [23]. Results from a recent prospective randomized control trial found that patients who underwent IOLT in RYGB had significantly lower rates of anastomotic leaks (0% vs. 8%) and a lower need for reoperation (0% vs. 8%) [24]. Another study with a large cohort by Haddad et al. reported an intraoperative leak rate of 3.5% (80 of 2,308 RYGB), with 46 patients undergoing intraoperative repair [25]. Postoperative leaks were detected in only four of these patients (0.2%) [25].

This evidence may highlight the important role IOLT can play in improving postoperative outcomes, particularly procedures such as RYGB. Early detection may allow surgeons to prevent more serious complications later, reducing both morbidity and healthcare costs. More studies are needed to examine these claims more thoroughly.

An interesting finding in a study by Jung et al. in 2021 was that, although postoperative leak rates after bariatric surgery were low irrespective of IOLT, patients who had IOLT in primary SG had lower postoperative bleeding rates [22]. In this propensity-matched analysis of 363,042 patients who underwent bariatric surgery, the impact of IOLT on postoperative outcomes was evaluated [22]. IOLT was performed in 82% of cases, more commonly in RYGB (93%) compared to SG (77%) [22]. Postoperative leak rates were low and similar between groups with and without IOLT, but IOLT in primary SG was associated with a significant reduction in postoperative bleeding (0.6% vs. 0.8%, p = 0.002) [22]. This was a small absolute reduction but represented a 25% relative risk reduction. Procedure times were longer in cases with IOLT, with a mean difference of about nine to 10 minutes in both primary RYGB and SG [22]. The authors concluded that IOLT may be recommended in primary SG due to the decreased risk of bleeding, but its primary role in preventing leaks was uncertain [22].

The reduction in bleeding highlights that IOLT may confer additional benefits beyond leak detection, particularly in surgeries like SG, where bleeding can pose significant postoperative risks. This effect could make IOLT more appealing in cases where bleeding risks are high. Future studies are needed to fully explore its potential in this area.

Claims have been made that IOE is underutilized in bariatric procedures despite its many benefits [21]. Several studies recommend its routine use in LSG and RYGB to decrease the risks of leaks, stenosis, and bleeding [25-27]. An analysis of the American College of Surgeons National Surgical Quality Improvement Program database by Minhem et al. from 2011 until 2016 found that out of 62,805 cases of SG and 50,047 cases of RYGB, 17.9% and 19.7% underwent IOE, respectively [21]. SG cases who had IOE were associated with a decrease in sepsis (0.37% vs. 0.21%, adjusted OR (AOR) = 0.55 (0.36, 0.84)), unplanned reoperation (0.58% vs. 0.38%, AOR = 0.61 (0.44, 0.85)), prolonged hospital stay (14.9% vs. 14.0%, AOR = 0.87 (0.82, 0.92)), and composite complications (1.43% vs. 1.17%, AOR = 0.78 (0.65, 0.94)) [21]. Large multicenter prospective studies are needed to validate these points, explore the benefits of IOE in this patient population, and study longer-term benefits. In the study by Haddad et al., the effectiveness of IOE in decreasing anastomotic complications in RYGB was evaluated [25]. In this retrospective study, an IOE was completed on 2,308 patients to evaluate the anastomosis and to also perform an IOLT with air insufflation [25]. A leak was detected by IOE in 3.5% of cases, and immediate repair was executed in 2% [25]. This technique led to low gastrojejunal anastomosis-related morbidity after RYGB within 90 days, with a leak rate of 0.2% and a stricture rate of 1.1% [25]. An important result is that only 0.1% of patients had an iatrogenic injury due to the IOE [25]. Although it praises the important benefits of IOE, this study is limited by its retrospective design and a lack of a control group who did not undergo IOE.

Although IOE may be effective in detecting anastomotic leaks and other complications, the lack of standardization across centers and the absence of larger prospective trials are also factors affecting its widespread adoption. Future studies should focus on addressing these gaps. It should be noted that while IOLT offers benefits, it should be viewed as part of a comprehensive surgical strategy and cannot replace meticulous surgical technique with careful postoperative monitoring.

Limitations of IOLT

IOLT is not without limitations. A recent retrospective study with a large cohort of patients who underwent IOLT routinely found that IOLT was associated with increased rates of postoperative leaks for both SG and RYGB [4]. Its routine use for SG has yielded a sensitivity of 0% in some series [18]. In a multicenter study of routine versus selective leak testing for SG by Bingham et al., it was found that routine IOLT had very poor sensitivity and was negative in 91% of patients who later developed postoperative leaks [28]. Moreover, it was found that the use of IOLT was not associated with a decrease in the incidence of leaks, and routine IOLT had no benefit over selective IOLT [28]. Theoretical concerns have been raised in the literature about the intraluminal shear stress exerted by these tests on the staple line or the anastomosis, which may result in perioperative staple line weakness and subsequent leak, which is especially concerning with the air leak test [29]. However, these concerns have been refuted in a study by Causey et al., which aimed to assess the burst pressure of fresh staple lines in SG and duodenal switch (DS) [29]. The study involved 30 patients (21 SG and 9 DS); IOLT was performed using air or saline, and burst pressure analysis was performed on the resected specimens ex vivo [29]. They found that the staple line burst pressures exceeded the pressure typically exerted during IOLT and that IOLT pressures were not high enough to cause a potential leak [29]. Nevertheless, this study had several limitations, including a small sample size, a simulated environment (ex vivo testing of specimens), and did not assess postoperative outcomes. Another issue is the tests’ inability to detect ischemic leaks that develop in the first days after surgery [30]. Additionally, the effectiveness of these tests may be compromised by improper technique, misinterpretation of results, iatrogenic injury, longer operative times, and increased resource utilization [23]. For methylene blue specifically, while it may be effective in detecting small leaks, it carries the risk of mild to severe allergic reactions, although these are rare [31]. It has also been reported to impair intraperitoneal wound healing [32].

The risks of IOE and early endoscopies on a fresh anastomosis or staple line have created hesitancy among those performing the procedure [33]. A newly formed anastomosis is still in the process of healing from the surgical trauma and is met with the combination of air insufflation, local endoscope-related trauma, and torque, which may theoretically lead to a perforation of this newly formed tissue [33]. Although several studies have demonstrated that IOE and early endoscopies are safe and should be performed when necessary [25,33-35].

Lastly, one should always consider that a negative IOLT could give a surgeon a false sense of security [36], and postoperative assessments should always be diligent, despite a negative IOLT.

Intraoperative identification of leak potential and evolving technologies

In addition to traditional methods, indocyanine green (ICG) fluorescence testing has gained significant attention for its ability to identify tissue ischemia, which can lead to leaks before they even develop, making it a powerful tool for real-time imaging of tissue perfusion and blood supply [37]. ICG is a fluorescent water-soluble dye that binds to plasma proteins; it has fluorescent properties in the near-infrared spectrum [38]. Depending on its target tissue, ICG can be administered intravenously, submucosally, or into the ureteral system [38]. ICG is used in colorectal anastomosis assessment, ureteral visualization, sentinel lymph node identification, lymphatic drainage patterns, skin flap blood supply, live resection guidance, and resection margins in esophagectomies and other upper gastrointestinal surgeries, as well as other evolving applications [38,39]. When gastrointestinal anastomoses are examined, ICG is injected, and its fluorescence can be visualized using a near-infrared camera, allowing surgeons to ensure adequate blood flow and identify ischemic areas, thereby reducing the risk of postoperative leaks [38].

ICG fluorescence testing has shown promise not only in identifying ischemia but also in real-time decision-making during surgery, making it a versatile tool. Its application across multiple gastrointestinal surgeries highlights its broad potential in improving intraoperative outcomes.

While ICG fluorescence testing is effective in identifying ischemia, several other factors contribute to the pathophysiology of leaks beyond ischemia [37]. Furthermore, a well-described drawback to ICG is that the flow estimate using ICG is influenced by the concentration of the contrast agent in the blood, which means larger vessels will generate a higher signal [40]. This difference in intensity could result from dye concentration variations rather than suspected ischemic processes [41]. Consequently, relying on these signal changes may provide misleading information to the surgeon [41]. It is also worth mentioning a report of a novel leak test involving a blend of both methylene blue and ICG [42]. In a prospective study by Hagen et al., 95 patients underwent robotic RYGB, and the effectiveness of combining methylene blue and ICG for intraoperative leak detection was evaluated, comparing them to the air leak test and methylene blue alone [42]. While no leaks were detected with the air or methylene blue tests (0%), the ICG-methylene blue blend detected leaks in 4.2% of patients (4 patients) [42]. No adverse postoperative events were reported, and the authors concluded that the ICG-methylene blue blend was more sensitive for detecting small anastomotic leaks than either air or methylene blue alone [42]. ICG fluorescence testing is a promising and valuable intraoperative tool, and additional research is necessary to explore its full capabilities.

Another method for the identification of leak potential intraoperatively is laser speckle contrast imaging (LSCI). LSCI, like ICG, provides real-time perfusion data in the operating room [43]. It creates a color-coded heatmap of blood flow by capturing the scattering of laser light on red blood cells [43]. Its advantages over ICG are the ability for repeated, on-demand use, no dye injections, and immediate visualization without the need to wait for dye transit [43]. In a study by Liu et al. at Stanford University, the utility of LSCI was compared to ICG fluorescence for perfusion detection in robotic-assisted and laparoscopic surgeries [43]. A total of 67 patients underwent elective surgery, including but not limited to colorectal and bariatric surgeries [43]. The study found that LSCI detected tissue perfusion with an accuracy of 97.5% in robotic cases and 100% in laparoscopic cases; no adverse events were reported in either group [43]. It concluded that LSCI was a feasible and safe alternative to ICG [43]. LSCI has demonstrated value, and more rigorous studies are needed to determine its full scope and applicability.

Hyperspectral imaging (HSI) has also emerged as a tool for intraoperative tissue perfusion assessment [44]. HSI is a noninvasive technique that captures and analyzes light reflected from tissue to assess oxygenation, hemoglobin content, and water index [44]. No contrast agents or ionizing radiation are required, and real-time assessment of tissue well-being can be established [45]. Studies are scarce regarding its use in RYGB and SG; however, literature is emerging regarding its potential uses in upper and lower gastrointestinal surgeries [41,44,45]. In a study by Zimmermann et al. in 2023, HSI was compared to visual inspection during esophagectomies with colon interposition to assess perfusion at the anastomosis [44]. Eight patients underwent the surgery, and HSI was used intraoperatively to measure perfusion at the base and tip of the colon conduit [44]. Only one patient (12.5%) experienced a postoperative leak requiring re-anastomosis on postoperative day 4, and HSI measurements showed a significant difference in perfusion between the base and tip of the colon conduit in this patient [44]. They concluded that HSI may be used as an objective tool for tissue perfusion assessment, but the study was limited by its small sample size, the absence of a control group, and short follow-up [44].

Jansen-Winkeln et al. compared HSI to ICG imaging in colorectal anastomoses [45]. In this study, both HSI and ICG were used to assess perfusion [45]. In 30 out of 32 patients, the imaging data from both methods were comparable, with ICG offering a clearer border zone and HSI providing a wider zone with more gradations in perfusion [45]. Because both methods were comparable, the advantage of HSI was that it was contact-free and did not require dye injections, whereas its drawback was that it had a wider border zone, making it less precise than ICG in some cases [45]. Other studies report high accuracy with HSI use during laparoscopic surgery, although its high cost and the need for special equipment were among its disadvantages [41]. These studies accentuate the growing and evolving nature of HSI as an intraoperative tool for tissue perfusion assessment. Further studies are necessary to explore its full capabilities and potential applications.

## Conclusions

IOLT remains a critical tool in bariatric surgery for detecting leaks early and preventing postoperative complications. Traditional methods such as air leak and methylene blue dye tests are widely used, although their effectiveness varies. Emerging technologies, including ICG fluorescence, LSCI, and HSI, offer promising alternatives for real-time tissue perfusion assessment, each with distinct advantages and limitations. While these advanced imaging techniques show potential in improving patient outcomes, their cost, technical complexity, and limited accessibility warrant further investigation. Larger, multicenter studies are needed to establish the long-term benefits of these technologies and optimize their application in clinical practice. Ultimately, a comprehensive approach combining traditional methods with evolving technologies may provide the best strategy for reducing complications in bariatric surgery.
